# In-vitro inhibition of IFNγ^+^ iTreg mediated by monoclonal antibodies against cell surface determinants essential for iTreg function

**DOI:** 10.1186/1471-2172-13-47

**Published:** 2012-08-21

**Authors:** Volker Daniel, Mahmoud Sadeghi, Haihao Wang, Gerhard Opelz

**Affiliations:** 1 Department of Transplantation-Immunology, Institute of Immunology, University of Heidelberg, Im Neuenheimer Feld 305, Heidelberg, 69120, Germany; 2 Institute of Organ Transplantation, Tongji Hospital, Huazhong University of Science and Technology, Wuhan, 430030, China

**Keywords:** IFNγ^+^ iTreg, IFNγ^+^Foxp3^+^, IFNγ^+^CD127^-^, CD178, CD152, CD279, CD28, CD95, HLA-DR, Inhibition, Cell proliferation

## Abstract

**Background:**

IFNγ-producing CD4^+^CD25^+^Foxp3^+^ PBL represent a subtype of iTreg that are associated with good long-term graft outcome in renal transplant recipients and suppress alloresponses in-vitro. To study the mechanism of immunosuppression, we attempted to block cell surface receptors and thereby inhibited the function of this iTreg subset in-vitro using monoclonal antibodies and recombinant proteins.

**Methods:**

PBL of healthy control individuals were stimulated polyclonally in-vitro in the presence of monoclonal antibodies or recombinant proteins against/of CD178, CD152, CD279, CD28, CD95, and HLA-DR. Induction of IFNγ^+^ iTreg and proliferation of effector cells was determined using four-color fluorescence flow cytometry. Blockade of iTreg function was analyzed using polyclonally stimulated co-cultures with separated CD4^+^CD25^+^CD127^-^IFNγ^+^ PBL.

**Results:**

High monoclonal antibody concentrations inhibited the induction of CD4^+^CD25^+^Foxp3^+^IFNγ^+^ PBL (anti-CD152, anti-CD279, anti-CD95: p < 0.05) and CD4^+^CD25^+^CD127^-^IFNγ^+^ PBL (anti-CD178, anti-CD152, anti-CD279, anti-CD95: p < 0.05). Effector cell proliferation increased with increasing antibody concentrations in culture medium (anti-CD178 and anti-CD279: p < 0.05). Conversely, high concentrations of recombinant proteins induced formation of CD4^+^CD25^+^Foxp3^+^IFNγ^+^ PBL (rCD152 and rCD95: p < 0.05) and decreased cell proliferation dose-dependently (rCD178 and rCD95: p < 0.05). Our data suggest an inverse association of iTreg induction with effector cell proliferation in cell culture which is dependent on the concentration of monoclonal antibodies against iTreg surface determinants. 3-day co-cultures of polyclonally stimulated PBL with separated CD4^+^CD25^+^CD127^-^IFNγ^+^ PBL showed lower cell proliferation than co-cultures with CD4^+^CD25^+^CD127^-^IFNγ^-^ PBL (p < 0.05). Cell proliferation increased strongly in CD4^+^CD25^+^CD127^-^IFNγ^-^ PBL-containing co-cultures in the presence of monoclonal antibody (anti-CD28, anti-CD152, anti-CD279: p < 0.05) but remained low in co-cultures with CD4^+^CD25^+^CD127^-^IFNγ^+^ PBL (with the exception anti-CD28 monoclonal antibody: p < 0.05). Monoclonal antibodies prevent iTreg induction in co-cultures with CD4^+^CD25^+^CD127^-^IFNγ^-^ PBL but do not efficiently block suppressive iTreg function in co-cultures with CD4^+^CD25^+^CD127^-^IFNγ^+^ PBL.

**Conclusions:**

CD178, CD152, CD279, CD28, CD95, and HLA-DR determinants are important for induction and suppressive function of IFNγ^+^ iTreg.

## Background

Recently, we reported that CD4^+^CD25^+^Foxp3^+^IFNγ^+^ PBL were more frequently detectable in renal transplant recipients with good than in recipients with impaired long-term graft function [1]. IFNγ-secreting CD4^+^CD25^+^Foxp3^+^ iTreg are usually undetectable in the peripheral blood of healthy individuals [1,2]. CD4^+^CD25^+^Foxp3^+^IFNγ^+^ PBL separated from primary MLC were shown to inhibit allogeneic secondary MLC responses mainly antigen-unspecifically, but in part also antigen-specifically [2-7]. Separated CD4^+^CD25^+^IFNγ^+^ co-expressed Foxp3, IL4, IL10, and TGFß intracellularly, suggesting that release of these immunosuppressive cytokines is involved in the immunosuppressive mechanism of IFNγ^+^ iTreg [2]. Using polyclonally stimulated cell cultures, we showed that CD4^+^CD25^+^Foxp3^+^, CD4^+^CD25^+^IFNγ^+^ and CD4^+^IFNγ^+^Foxp3^+^ PBL co-expressed CD178, CD28, CD95, HLA-DR, CD152, and CD279 [4]. Our current work addresses the hypothesis that these cell surface determinants, representing receptors involved in cell-cell interactions, contribute to immunosuppression mediated by this particular iTreg subset. To address this question, we attempted to block these cell surface receptors and their ligands using monoclonal antibodies and recombinant proteins. We studied two different subsets of IFNγ-secreting iTreg subsets, namely CD4^+^CD25^+^Foxp3^+^IFNγ^+^ and CD4^+^CD25^+^CD127^-^IFNγ^+^ iTreg. Because it was shown that CD4^+^CD25^+^Foxp3^+^ and CD4^+^CD25^+^CD127^-^ iTreg subsets overlap and represent in part different iTreg subpopulations [8], we investigated both subsets and particular those cells that produce intracellular IFNγ.

## Results

We studied whether monoclonal antibodies or recombinant proteins reactive with cell surface determinants affect induction of CD4^+^CD25^+^Foxp3^+^IFNγ^+^ and CD4^+^CD25^+^CD127^-^IFNγ^+^ PBL or cell proliferation in-vitro. PBL of 5 healthy control individuals (HC1-HC5) were separated from heparinized whole blood and stimulated for 16 h using PMA/Ionomycin (iTreg induction) or for 3 days using PHA (cell proliferation) in the presence of monoclonal antibodies against, or recombinant proteins of, CD28, CD95, CD152, CD178, CD278, and HLA-DR. CD4^+^CD25^+^Foxp3^+^IFNγ^+^ and CD4^+^CD25^+^CD127^-^IFNγ^+^ PBL as well as proportions of proliferating lymphoblasts with low CFSE staining were determined.

### Monoclonal antibodies against CD28, CD95, CD152, CD178, CD278, and HLA-DR

**Figure 1:**
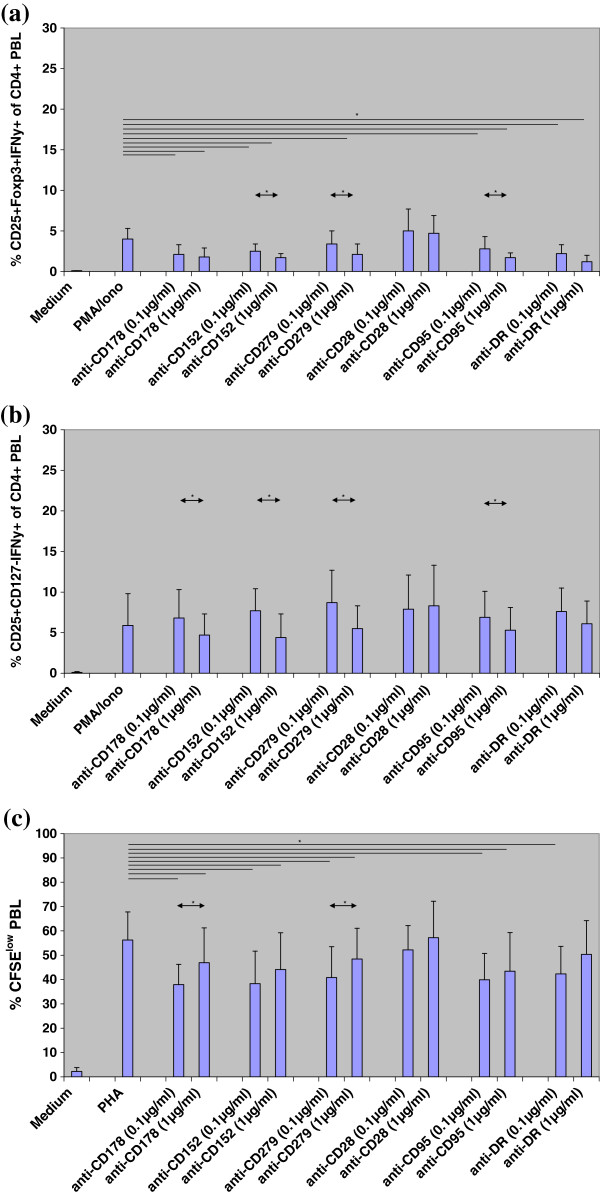
**Induction of CD4**^**+**^**CD25**^**+**^**Foxp3**^**+**^**IFNγ**^**+**^**and CD4**^**+**^**CD25**^**+**^**CD127**^**-**^**IFNγ**^**+**^**PBL and cell proliferation in the presence of PMA/Ionomycin and monoclonal antibodies against cell surface molecules.** (**a**, **b**) PBL of 5 healthy control individuals (HC1-HC5) were incubated in medium or stimulated with PMA/Ionomycin for 16 h in the presence of monoclonal antibodies against CD178, CD152, CD279, CD28, CD95, and HLA-DR in final concentrations of 0.1 μg/ml and 1 μg/ml. The assay was measured using four-color fluorescence flow cytometry. Compared to PMA-Ionomycin-stimulated cell cultures without monoclonal antibody, addition of anti-CD178, anti-CD152, anti-CD279, anti-CD95, and anti-HLA-DR monoclonal antibody blocked induction of CD4^+^CD25^+^Foxp3^+^IFNγ^+^ PBL (p < 0.05) but not induction of CD4^+^CD25^+^CD127^-^IFNγ^+^ PBL (p = n.s.). When cell cultures with a 1-log difference in antibody concentration were compared, higher concentrations of anti-CD152, anti-CD279, and anti-95 monoclonal antibody blocked induction of CD4^+^CD25^+^Foxp3^+^IFNγ^+^ PBL (p < 0.05) whereas higher anti-CD178, anti-CD152, anti-CD279, and anti-CD95 concentrations blocked induction of CD4^+^CD25^+^CD127^-^IFNγ^+^ PBL (p < 0.05) compared to lower concentrations of these antibodies in the cell cultures. (**c**) PBL of 5 healthy control individuals (HC1-HC5) were incubated in medium or stimulated with PHA for 3 days in the presence of monoclonal antibodies against CD178, CD152, CD279, CD28, CD95, and HLA-DR in final concentrations of 0.1 μg/ml and 1 μg/ml. The proportion of CFSE^low^ PBL was determined using flow cytometry. With the exception of anti-CD28, all monoclonal antibodies reduced cell proliferation (all p < 0.05) and cell proliferation tend to normalize in the presence of high antibody concentrations, especially of anti-CD178 and anti-CD279 (p < 0.05). In the figure, statistical significant differences of assays with vs without monoclonal antibodies are indicated by lines, whereas statistical significant differences of assays with low vs high monoclonal antibody concentrations are indicated by arrows. Data are given as mean±SD. *p < 0.05.

Anti-CD178, anti-CD152, anti-CD279, anti-CD95, and anti-HLA-DR but not anti-CD28 monoclonal antibody inhibited the induction of CD4^+^CD25^+^Foxp3^+^IFNγ^+^ PBL as compared to cell cultures without monoclonal antibody (all p < 0.05). Inhibition was dose-dependent and increased in parallel with antibody concentration in the cell culture (CD4^+^CD25^+^Foxp3^+^IFNγ^+^ PBL: anti-CD152, anti-CD279, and anti-CD95: all p < 0.05; CD4^+^CD25^+^CD127^-^IFNγ^+^ PBL: anti-178, anti-CD152, anti-CD279, and anti-CD95: all p < 0.05) (Figures 1a, b). Conversely, cell proliferation was lower in cell cultures with than in cultures without monoclonal antibody (p < 0.05; exception: anti-CD28) (Figure 1c). Cell proliferation increased with increasing antibody concentration in culture (anti-CD178 and anti-CD279: p < 0.05; anti-CD152 and anti-DR: p = 0.080). It appears that monoclonal antibody blocks iTreg induction and function dose-dependently and abrogates inhibition of cell proliferation in culture.

### Recombinant CD28, CD95, CD152, CD178, and CD278

**Figure 2:**
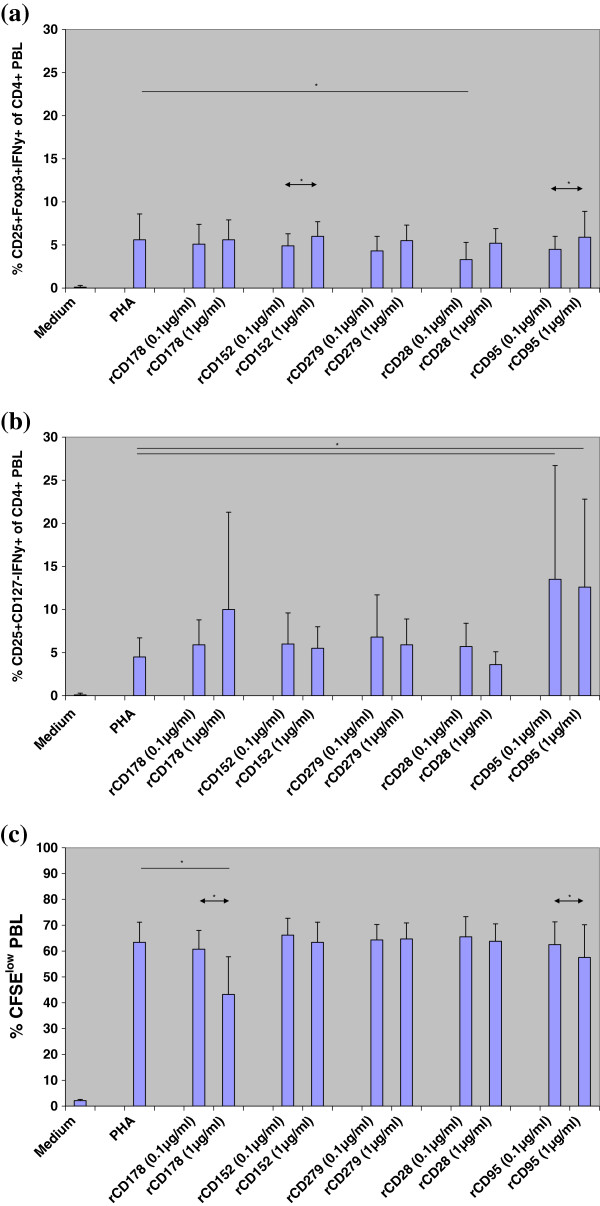
**Induction of CD4**^**+**^**CD25**^**+**^**Foxp3**^**+**^**IFNγ**^**+**^**and CD4**^**+**^**CD25**^**+**^**CD127**^**-**^**IFNγ**^**+**^**PBL and cell proliferation in the presence of PMA/Ionomycin and recombinant proteins against cell surface molecules.** (**a**, **b**) PBL of 5 healthy control individuals (HC1-HC5) were incubated in medium or stimulated with PMA/Ionomycin for 16 h in the presence of recombinant rCD178, rCD152, rCD279, rCD28, and rCD95 in final concentrations of 0.1 μg/ml and 1 μg/ml. Then, assay was measured using four-color fluorescence flow cytometry. Proportions of CD4^+^CD25^+^Foxp3^+^IFNγ^+^ and CD4^+^CD25^+^CD127^-^IFNγ^+^ PBL were calculated as proportion of all cultured CD4^+^ PBL. rCD28 reduced induction of CD4^+^CD25^+^Foxp3^+^IFNγ^+^ PBL (p < 0.05). Higher concentrations of recombinant proteins tend to increase iTreg induction, especially of rCD152 and rCD95 (p < 0.05). In the presence of rCD95, CD4^+^CD25^+^CD127^-^IFNγ^+^ PBL increased strongly (with vs without rCD95: p < 0.05). (**c**) PBL of 5 healthy control individuals (HC1-HC5) were incubated in medium or stimulated with PHA for 3 days in the presence of recombinant rCD178, rCD152, rCD279, rCD28, and rCD95 in final concentrations of 0.1 μg/ml and 1 μg/ml. Then, proportion of CFSE^low^ PBL was determined using flow cytometry. rCD178 and rCD95 reduced cell proliferation strongly (p < 0.05). Data are given as mean±SD.

Exposure to recombinant proteins induced the formation of CD4^+^CD25^+^Foxp3^+^IFNγ^+^ PBL (rCD152 and rCD95: p < 0.05; rCD28: p = 0.080) (Figures 2a, b) and was associated with decreased cell proliferation dose-dependently (rCD178 and rCD95: p < 0.05) (Figure 2c).

Next, we tried to block the suppressive function of separated pre-activated CD4^+^CD25^+^CD127^-^IFNγ^+^ PBL in polyclonally stimulated co-cultures using monoclonal antibodies against cell surface determinants of separated IFNγ^+^ iTreg.

### Co-cultures with separated CD4^+^CD25^+^CD127^-^IFNγ^+^ PBL in the presence of monoclonal antibodies against CD28, CD95, CD152, CD178, CD278, and HLA-DR

**Figure 3:**
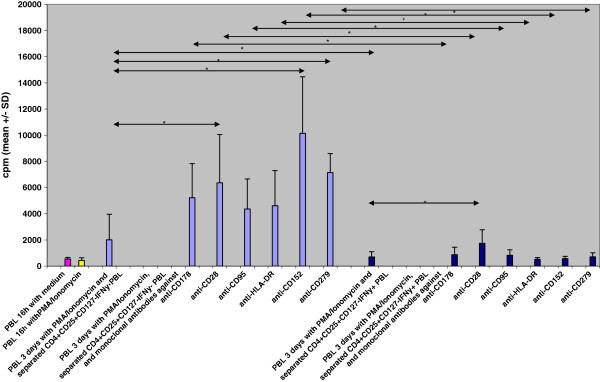
**Functional inhibition of iTreg by monoclonal antibodies against cell surface molecules.** PBL of 9 healthy control individuals were separated from heparinized whole blood and incubated for 16 h with medium or PMA/Ionomycin. Then, CD4^+^CD25^+^CD127^-^IFNγ^+^ PBL were separated from CD4^+^CD25^+^CD127^-^IFNγ^-^ PBL and both cell fractions were added to autologous PBL that were stimulated with PMA/Ionomycin for another 3 days in the absence or presence of monoclonal antibodies against cell surface molecules of iTreg such as CD178, CD152, CD279, CD28, CD95, and HLA-DR. Cell proliferation was measured as counts per minute (cpm) of ^3^ H-thymidine incorporation. All co-cultures with separated CD4^+^CD25^+^CD127^-^IFNγ^+^ PBL showed lower cell proliferation than co-cultures with separated CD4^+^CD25^+^CD127^-^IFNγ^-^ PBL (p < 0.05). Addition of monoclonal antibodies against CD28, CD152 and CD279 increased cell proliferation in cell cultures containing CD4^+^CD25^+^CD127^-^IFNγ^-^ PBL (p < 0.05). Of cell cultures with CD4^+^CD25^+^CD127^-^IFNγ^+^ PBL, only those with CD28 monoclonal antibody showed a slightly increased cell proliferation (p < 0.05). Data are given as mean±SD. *p < 0.05.

PBL of 9 healthy controls (HC1-HC9) were separated from heparinized whole blood and stimulated for 16 h using PMA/Ionomycin. CD4^+^CD25^+^CD127^-^IFNγ^+^ PBL were separated from CD4^+^CD25^+^CD127^-^IFNγ^-^ PBL and each of the two cell fractions was added to co-cultures of autologous PBL stimulated with PMA/Ionomycin for another 3 days in the presence or absence of monoclonal antibodies against CD28, CD95, HLA-DR, CD152, CD178, or CD279 (Figure 3).

3-day co-cultures with separated CD4^+^CD25^+^CD127^-^IFNγ^+^ PBL showed lower cell proliferation than co-cultures with CD4^+^CD25^+^CD127^-^IFNγ^-^ PBL (p = 0.011). Cell proliferation increased in CD4^+^CD25^+^CD127^-^IFNγ^-^ PBL-containing co-cultures in the presence of anti-CD28, anti-CD152, or anti-CD279 monoclonal antibody (with vs without mab: CD28 p = 0.015, CD152 p = 0.028, CD279 p = 0.046, CD178 p = 0.066, CD95 p = 0.066, and HLA-DR p = n.s.) whereas in co-cultures with CD4^+^CD25^+^CD127^-^IFNγ^+^ PBL cell proliferation was consistently low, with the exception of co-cultures with anti-CD28 monoclonal antibody which showed increased proliferation (p = 0.011). Notably, all cultures with CD4^+^CD25^+^CD127^-^IFNγ^+^ PBL showed lower rates of cell proliferation than the corresponding cell cultures with CD4^+^CD25^+^CD127^-^IFNγ^-^ PBL (CD178 p = 0.008, HLA-DR p = 0.008, CD28 p = 0.015, CD95 p = 0.015, CD152 p = 0.028, CD279 p = 0.028) (Figure 3). It appears that monoclonal antibodies prevented iTreg induction in co-cultures with CD4^+^CD25^+^CD127^-^IFNγ^-^ PBL, allowing strong effector cell proliferation, whereas monoclonal antibodies did not block suppressive iTreg function efficiently in CD4^+^CD25^+^CD127^-^IFNγ^+^ PBL-containing co-cultures, resulting in no or only moderate increases of effector cell proliferation.

**Figure 4:**
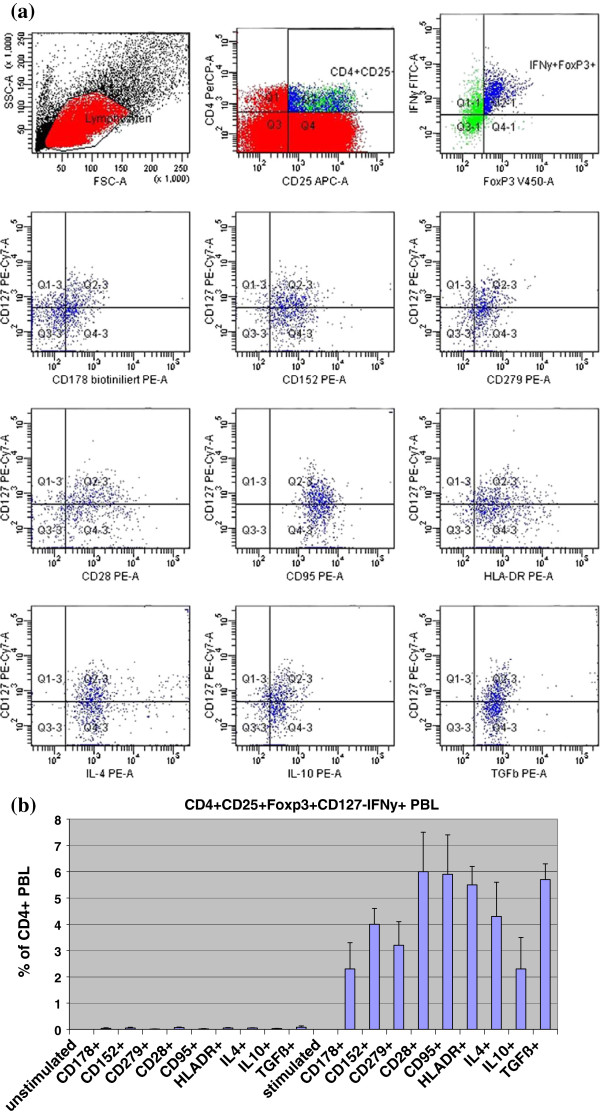
**Co-expression of CD178, CD152, CD279, CD28, CD95, HLA-DR, IL4, IL10, and TGFß on/in CD4**^**+**^**CD25**^**+**^**Foxp3**^**+**^**CD127**^**-**^**IFNγ**^**+**^**iTreg.** PBL of 3 healthy control individuals were stimulated for 16 h using PMA/Ionomycin. Lymphocytes were determined in a FSC/SSC dot plot using six-color-fluorescence flow cytometry. Gates for fluorochrome-labeled monoclonal antibodies were defined using appropriate isotype controls that were adjusted to <1% positive cells in the corresponding quadrants. Based on these isotype controls, CD4^+^CD25^+^Foxp3^+^CD127^-^IFNγ^+^ PBL were determined and co-expression of CD178, CD152, CD279, CD28, CD95, HLA-DR, IL4, IL10, and TGFß was analyzed. Figure (**a**) depicts the gating strategy, Figure (**b**) summarizes the results of the 3 healthy controls. Data are given as mean±SD. Unstim = incubated in medium, stimulated = incubated in PMA/Ionomycin.

**Figure 5:**
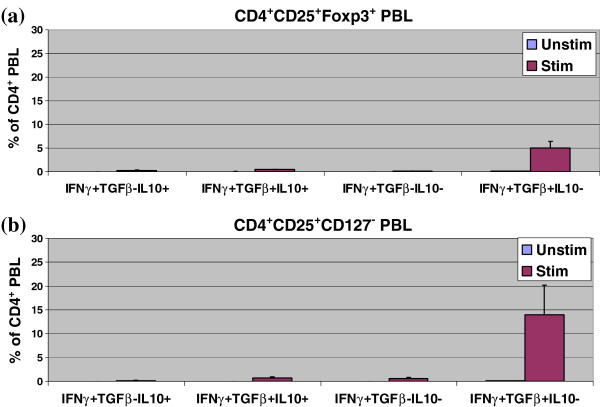
**Simultaneous co-expression of TGFß and IL10 in CD4**^**+**^**CD25**^**+**^**IFNγ**^**+**^**Foxp3**^**+**^**and CD4**^**+**^**CD25**^**+**^**IFNγ**^**+**^**CD127**^**-**^**iTreg.** PBL of 3 healthy control individuals were stimulated for 16 h using PMA/Ionomycin. Simultaneous co-expression of TGFß and IL10 in CD4^+^CD25^+^IFNγ^+^Foxp3^+^ and CD4^+^CD25^+^IFNγ^+^CD127^-^ iTreg was determined using six-color flow cytometry. (**a**, **b**) 0.5% of CD4^+^CD25^+^IFNγ^+^Foxp3^+^ and 0.7% of CD4^+^CD25^+^IFNγ^+^CD127^-^ iTreg co-expressed the immunosuppressive cytokines TGFß as well as IL10 intracellularly, and 5% of CD4^+^CD25^+^IFNγ^+^Foxp3^+^ and 14% of CD4^+^CD25^+^IFNγ^+^CD127^-^ iTreg produced TGFß but not IL10 (all percentages with respect to total of CD4^+^ PBL in the cell culture). Data are given as mean±SD. Unstim = incubated in medium, stimulated = incubated in PMA/Ionomycin.

In a separate experiment with PBL stimulated for 16 h using PMA/Ionomycin, CD4^+^CD25^+^Foxp3^+^CD127^-^IFNγ^+^ PBL were shown to co-express CD178, CD152, CD279, CD28, CD95, HLA-DR, IL4, IL10, and TGFß (Figure 4). The data suggest that (a) CD4^+^CD25^+^Foxp3^+^CD127^-^IFNγ^+^ PBL co-express the corresponding target structure for the monoclonal antibodies and (b) that CD4^+^CD25^+^Foxp3^+^CD127^-^IFNγ^+^ PBL might exert immunosuppression by both cell-cell contact and secretion of immunosuppressive cytokines. Figure 5 shows that approximately 0.5% of CD4^+^CD25^+^IFNγ^+^Foxp3^+^ and 0.7% of CD4^+^CD25^+^IFNγ^+^CD127^-^ iTreg co-expressed simultaneously the immunosuppressive cytokines TGFß as well as IL10 intracellularly, and 5% of CD4^+^CD25^+^IFNγ^+^Foxp3^+^ and 14% of CD4^+^CD25^+^IFNγ^+^CD127^-^ iTreg produced TGFß but not IL10 (all percentages with respect to total of CD4^+^ PBL in the cell culture).

## Discussion

In previous experiments, we were able to induce CD4^+^CD25^+^Foxp3^+^IFNγ^+^ PBL by PMA/Ionomycin stimulation in-vitro and these iTreg were shown to suppress activation of responder cells in secondary MLC mainly antigen-unspecifically, although the strongest suppression was seen in antigen-specific settings [2,3]. Induction of CD4^+^CD25^+^Foxp3^+^IFNγ^+^ PBL peaked at 6 h of PMA/Ionomycin stimulation and decreased thereafter gradually [2]. Furthermore, we were able to show co-expression of CD178, CD28, CD95, HLA-DR, CD152, and CD279 on polyclonally stimulated CD4^+^CD25^+^Foxp3^+^, CD4^+^CD25^+^IFNγ^+^ and CD4^+^Foxp3^+^IFNγ^+^ PBL [4]. We hypothesized that interaction of at least some of these cell surface receptors with their ligands might contribute to immunosuppressive function. In the present study, we studied whether blocking of these receptors affects induction and immunosuppressive function of IFNγ^+^ iTreg in-vitro.

We found that induction of IFNγ^+^ iTreg was prevented in the presence of monoclonal antibodies against CD28, CD95, CD152, CD178, CD278, and HLA-DR, whereas recombinant proteins of these determinants induced IFNγ^+^ iTreg. The frequency of IFNγ^+^ iTreg was inversely associated with the cell proliferation rate during 3-day cell culture. When CD4^+^CD25^+^CD127^-^IFNγ^+^ PBL were separated and added to autologous PBL, they prevented cell proliferation; in contrast, addition of separated CD4^+^CD25^+^CD127^-^IFNγ^-^ PBL increased proliferation in cell co-cultures. Monoclonal antibodies increased cell proliferation more markedly in cell cultures containing CD4^+^CD25^+^CD127^-^IFNγ^-^ PBL than in cell cultures containing CD4^+^CD25^+^CD127^-^IFNγ^+^ PBL. It thus appears that monoclonal antibodies prevented iTreg induction in co-cultures with separated CD4^+^CD25^+^CD127^-^IFNγ^-^ PBL, allowing strong effector cell proliferation, whereas monoclonal antibodies did not block suppressive iTreg function efficiently in co-cultures containing separated CD4^+^CD25^+^CD127^-^IFNγ^+^ PBL, resulting in no or only moderate increases of effector cell proliferation. In previously published experiments with secondary MLC and CFSE-stained responder cells, addition of CD4^+^CD25^+^IFNγ^+^ PBL separated from primary MLC inhibited the responder cell proliferation, determined as CFSE^low^ responder cells, stronger than CD4^+^CD25^+^IFNγ^-^ PBL remaining after separation, thus substantiating the immunosuppressive capacity of CD4^+^CD25^+^IFNγ^+^ PBL [2]. Interestingly, we observed a trend that cell cultures containing anti-CD28 monoclonal antibody showed increased induction of CD4^+^CD25^+^Foxp3^+^IFNγ^+^ and CD4^+^CD25^+^CD127^–^IFNγ^+^ PBL (Figure 1a, b), increased proliferation of CFSE-labelled responder cells (Figure 1c), and increased activation/proliferation when co-cultured with CD4^+^CD25^+^Foxp3^+^IFNγ^+^ PBL (Figure 3) compared to cell cultures with other monoclonal antibodies. We speculate that anti-CD28 monoclonal antibody amplifies cell activation in polyclonally stimulated cell cultures and induces proliferation of both IFNγ-secreting CD4^+^CD25^+^Foxp3^+^/ CD4^+^CD25^+^CD127^-^ PBL (Figures 1a, c) as well as responder T cells (Figure 1c, 3). In summary, CD178, CD152, CD279, CD28, CD95, and HLA-DR determinants were shown to be important for induction and function of IFNγ^+^ iTreg. Blockade of CD178, CD152, CD279, CD95, and HLA-DR determinants prevents induction of IFNγ-producing iTreg during polyclonal stimulation and induces effector cell proliferation dose-dependently. Recombinant proteins show the reverse effect. It therefore can be concluded that interaction of CD178, CD152, CD279, CD28, CD95, and HLA-DR determinants on CD4^+^CD25^+^Foxp3^+^IFNγ^+^ and CD4^+^CD25^+^CD127^-^IFNγ^+^ PBL are essential for the suppressive function of these IFNγ-producing iTreg subsets.

Our findings are in line with the findings of other investigators. Strong CD28 co-stimulation suppressed induction of Treg from naïve precursors through Lck signaling and provided a rational for promoting T-cell immunity or tolerance by regulating Treg through targeting CD28 signaling [9]. CD152 (CTLA-4) controls homeostasis and suppressive capacity of regulatory T cells in mice [10]. Blockade of CD152 signaling resulted in impairment of the suppressive capacity of Treg [10]. Expression of CD152 on Treg serves to control T cell proliferation, to confer resistance against activation-induced cell death, and to maintain the suppressive function of Treg [10]. Distinctive characteristics of PD-1 expression on peripheral CD4^+^CD127^low^CD25^high^Foxp3^+^ Treg in chronic HCV infection were associated with impaired adaptive immunity as well as viral long-term persistence [11]. In a mouse model, administration of CD279 monoclonal antibody CT-011 prolonged Treg inhibition induced by low-dose cyclophosphamide, leading to a sustainable synergistic decrease of splenic and tumor-infiltrated Treg [12]. This strategy led to complete regression of established tumors in a significant percentage of treated animals, with survival prolongation [12]. Human Treg express Fas as well as FasL and either kill activated T effector lymphocytes and thereby induce immunosuppression, or vice versa, are killed by T effector cells and thereby eliminate immunosuppression [13-16]. A recently published study showed that Foxp3 negatively regulated CD95L expression in Treg and demonstrated that Treg are susceptible to homeostatic control by CD95 stimulation [17]. Our own studies show that CD4^+^CD25^+^Foxp3^+^IFNγ^+^CD178^+^ human iTreg are generated during polyclonal stimulation in-vitro and that they suppress alloresponses by apoptosis of responder cells [4]. Others showed that a distinct subset of HLA-DR^+^-regulatory T cells is involved in the induction of preterm labor during pregnancy and in the induction of organ rejection after transplantation [18].

Because CD4^+^CD25^+^Foxp3^+^ and CD4^+^CD25^+^CD127^-^ iTreg subsets overlap by two-thirds and represent in part different iTreg subpopulations [8], we investigated both subsets and particular those cells that produce intracellular IFNγ. It was reported that differences in CD4^+^CD25^+^Foxp3^+^ and CD4^+^CD25^+^CD127^-^ iTreg frequencies exist in the blood of patients with systemic scleroderma [8]. Recently, Foxp3^-^ CD4^+^CD25^+^CD127^-^ iTreg with appreciable suppressive activity on effector T cell proliferation, although less than that displayed by Treg cells from healthy controls, were demonstrated in patients with immune dysregulation, polyendocrinopathy, enteropathy, X-linked (IPEX) syndrome [19]. Our data show a similar reaction pattern of both iTreg subsets in the presence of monoclonal antibodies against the studied cell determinants (Figure 1), suggesting that both subsets co-express CD178, CD152, CD28, CD95, and HLA-DR (Figure 4). However, assays with recombinant protein exhibit in part divergent results of the two iTreg populations (Figure 2).

We investigated the induction of IFNγ^+^ iTreg in-vitro. This particular iTreg subset represents the first line of iTreg during an immune response because they express IFNγ receptors, are induced by IFNγ, and represent sensors for immediate immune responses [2]. IFNγ-producing CD4^+^CD25^+^Foxp3^+^ PBL were associated with good long-term graft outcome in renal transplant recipients and suppressed alloresponses in-vitro [1,2]. Others reported similar findings in mice. Mouse iTreg generated by IFNγ-conditioning of non-regulatory CD4^+^ T cells in the presence of alloantigen not only prevented the acute rejection of skin and islet allografts, but also the development of chronic allograft dysfunction (CAD)-associated vasculopathy of an arterial transplant [20-23].

## Conclusion

Our data suggest that CD28, CD95, CD152, CD178, CD278, and HLA-DR interactions are necessary for the induction and suppressive function of IFNγ^+^ iTreg.

## Methods

### Healthy controls

Laboratory staff served as healthy controls. All controls gave informed consent for the tests performed within this study and the study was approved by the local ethical committee “Ethikkommission, Medizinische Fakultät Heidelberg”. The study was conducted in adherence to the Declaration of Helsinki.

### Polyclonal stimulation of PBL

PBL were separated from heparinized whole blood by Ficoll density gradient centrifugation and stimulated for 16 h using a mixture of phorbol 12-myristate 13-acetate (PMA; final concentration in medium: 10 ng/ml; Sigma Aldrich, Munich, Germany) and ionomycin (1 μg/ml; Sigma Aldrich, Munich, Germany), or for 3 days using either PMA/Ionomycin or phytohaemagglutinin (PHA, final concentration in medium: 10 ng/ml; Sigma Aldrich, Munich, Germany) in RPMI medium containing 10% FCS, L-Glutamin, and Penicillin/ Streptomycin (all from Invitrogen Gibco, Paisley, Scotland) as described previously [2]. All tests were done in triplicate. The mean of triplicate assays was calculated and used for statistical analysis. Cell activation and proliferation was determined using ^3^ H-thymidine incorporation. Alternatively, cell proliferation was measured using CFSE (CellTrace CFSE Cell Proliferation Kit, Invitrogen) staining according to the manufacturer's instruction [2].

### Separation of CD4^+^CD25^+^CD127^-^IFNγ^+^ PBL from PMA/Ionomycin-stimulated short-term cell cultures

As described previously, CD4^+^ PBL were separated by negative selection using biotinylated monoclonal antibodies against CD14, CD16, CD56, CD123, CD36, CD8, CD19, glycophorin (Dynabeads Regulatory CD4^+^CD25^+^ T cell kit, Invitrogen, Dynal Oslo, Norway), CD127 (BD Biosciences, Heidelberg, Germany), and streptavidin-coupled beads according to the manufacturer's instructions [2]. CD4^+^CD127^-^ PBL were incubated with bead-coupled CD25 monoclonal antibodies and CD4^+^CD127^-^CD25^+^ PBL were separated using a magnet. CD25 monoclonal antibodies were removed from the cell surface by addition of anti-Fab antibody (DETACHaBEAD, Invitrogen). CD4^+^CD25^+^CD127^-^ PBL were incubated with a biotinylated IFNγ monoclonal antibody (BD Biosciences, Heidelberg, Germany, clone 4S.B3) (20 μl antibody to 500 μl cell suspension) for 20 min. Thereafter, CD4^+^CD25^+^CD127^-^IFNγ^+^ PBL were separated from CD4^+^CD25^+^CD127^-^IFNγ^-^ PBL using streptavidin-coupled beads (Dynabeads Biotin Binder, Invitrogen). Separated PBL were added to co-cultures of autologous PBL stimulated for another 3 days with PMA/Ionomycin in the presence or absence of monoclonal antibodies against cell surface molecules.

### Co-cultures of PMA/Ionomycin-stimulated PBL with autologous separated CD4^+^CD25^+^CD127^-^IFNγ^+^ and CD4^+^CD25^+^CD127^-^IFNγ^-^ PBL in the presence or absence of monoclonal antibodies

10^6^ PBL (100 μl) were co-cultured with 10^5^ separated CD4^+^CD25^+^CD127^-^IFNγ^+^ or CD4^+^CD25^+^CD127^-^IFNγ^-^ PBL (100 μl). Monoclonal antibody was added to the co-cultures (final concentration 1 μg/ml). At the end of the culture period, PBL were pulsed with 20 μl of ^3^ H-thymidine (1 mCi/ml) per well for 16 h. Cells were harvested and ^3^ H-thymidine incorporation was measured using a ß-counter.

### Determination of PBL subsets

PBL subsets were determined as described previously [1]. For analysis of determinants on the cell surface, PBL were incubated with fluorochrome-labelled monoclonal antibodies against CD4, CD25, CD127, CD178, CD152, CD279, CD28, CD95, and HLA-DR (all from BD Biosciences). Intracellular determinants/cytokines were stained with fluorochrome-labelled monoclonal antibodies against Foxp3, IFNγ (clone B27), IL4, IL10 (all from BD Biosciences), and TGFß (R&D systems, Wiesbaden, Germany). Briefly, PBL were incubated with combinations of monoclonal antibodies for 30 min as described. Multi-color fluorescence was analyzed using a FACScalibur dual-laser, a FACScanto triple-laser, or a LSR II four-laser flow-cytometer (all BD Biosciences) [1]. When, in addition, intracellular proteins were studied, cell membranes were permeabilized using BD Perm/Wash buffer (BD Biosciences). At least 100,000 events were analyzed in the initial FSC/SSC dot plot. IFNγ monoclonal antibody used for cell separation (BD clone 4S.B3) and IFNγ monoclonal antibody used for cell staining (BD clone B27) were not competitive (data not shown) [2].

### Statistics

For statistical analysis, PASW Statistics program version 18 (IBM, Chicago, Illinois, USA) and Wilcoxon signed rank test were used. P-values of less than 0.05 were considered significant.

## Competing interests

In the past five years I did not receive reimbursements, fees, funding, or salary from an organization that may in any way gain or lose financially from the publication of this manuscript, either now or in the future. I do not hold any stocks or shares in an organization that may in any way gain or lose financially from the publication of this manuscript, either now or in the future.

## Authors’ contributions

VD designed the study and wrote the manuscript. GO made contributions to conception and design as well as analysis and interpretation of data. HW and MS have been involved in drafting the manuscript and revising it critically for important intellectual content. All authors have given final approval of the version to be published.
